# Cyst of the back of the hand

**DOI:** 10.11604/pamj.2017.28.52.13021

**Published:** 2017-09-20

**Authors:** Amina Kissou, Badr Eddine Hassam

**Affiliations:** 1 Service de Dermatologie, Centre Hospitalier Universitaire, Rabat, Maroc

**Keywords:** Cyst, epidermoid, hand

## Image in medicine

A 70 year-old man, farmer by profession consulted for a firm and painless nodule of the back of his right hand that was evolving since 09 years. The clinical examination revealed a subcutaneous nodular lesion; firm; immobile and painless. The diameter was about 0.8cm. The skin above was normal. Surgical excision was indicated. Intraoperatively the lesion was cystic and surrounded by a whitish membrane. Histological examination confirmed an epidermoid cyst who was abducted in totality. The occurrence of epidermoid cyst in hands can be explained by repeated trauma. It is seen most often in men “a manual worker.” Clinically, it is about a firm nodule often sitting at the distal phalanx in size from 0.5 to 2cm. The Pulp location is the common location against in back of the hand which is possible but unusual, as in our patient. The overlying skin is normal or punctuated and the lesion is painless in half of the cases. Treatment is the complete surgical excision of the capsule and the overlying skin to avoid the risk of recurrence.

**Figure 1:**
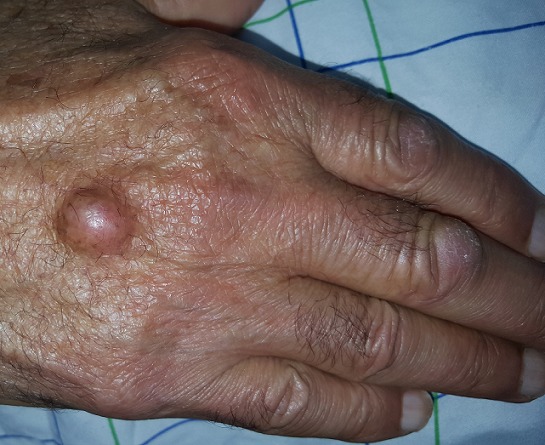
Cyct of the back of the hand

